# Reducing the burden of diabetes through better care across the disease continuum

**DOI:** 10.1002/ctm2.70897

**Published:** 2026-11-14

**Authors:** Siqi Yang, Till Winfried Bärnighausen, Zhong Cao

**Affiliations:** ^1^ Department of Radiology Nantong First People's Hospital, Affiliated Nantong Clinical College of Nantong University, Nantong University Nantong Jiangsu People's Republic of China; ^2^ Heidelberg Institute of Global Health, Universitatsklinikum Heidelberg Heidelberg Germany

1

The burden of diabetes extends beyond hyperglycaemia to complications, premature mortality, disability, and long‐term care. In 2024, approximately 589 million adults aged 20–79 years had diabetes, about one in nine in this age group; this figure may reach 853 million by 2050.^1^ Cardiovascular and kidney disease, visual loss, neuropathy, foot disease, and amputation impose substantial costs on patients and society. Chen and colleagues estimated cumulative global macroeconomic losses of INT$10.2 trillion during 2020–2050, excluding informal caregiving; including it increased the estimate, although uncertainty was substantial.^2^ These losses reflect the combined effects of clinical events, disability, treatment needs, and unpaid care across patients, families, health systems, and economies. Because the burden accumulates over time, earlier intervention, treatment beyond glycaemic targets, and sustained management are essential to interrupt these pathways.

Prevention and early diagnosis are complementary. For people at high risk of type 2 diabetes, structured lifestyle interventions, including healthy diets, physical activity, and weight management, can delay onset. Risk‐based testing complements lifestyle prevention and can identify diabetes before prolonged untreated disease causes complications.^3^ However, screening improves outcomes only when positive results lead to confirmatory diagnosis, accessible treatment, and sustained follow‐up. Earlier detection also offers opportunities in type 1 diabetes. Islet‐autoantibody testing can identify presymptomatic disease, particularly among people with familial or genetic risk, while metabolic staging identifies those requiring monitoring or preventive treatment.^4^ In a randomised trial involving high‐risk relatives with stage 2 type 1 diabetes, a 14‐day course of teplizumab extended median time to clinical diagnosis from 24.4 to 48.4 months.^5^ This finding demonstrates that teplizumab can delay clinical onset, not that it offers a cure. The trial was relatively small and enrolled a selected high‐risk population, so its findings should not be generalised to everyone at risk of type 1 diabetes. Importantly, presymptomatic diagnosis creates a window for disease‐modifying intervention rather than simply identifying disease sooner. However, implementation requires reliable screening pathways, specialist capacity, safety monitoring, and equitable access to care.

Diabetes treatment should improve cardiovascular and kidney outcomes, body weight, and quality of life while preventing complications and premature death. Prescribing must consider cardiovascular and kidney risk, hypoglycaemia, comorbidities, and patient preferences, alongside glycaemic control. Randomised outcome trials support the importance of this approach. In people with type 2 diabetes and established cardiovascular disease, empagliflozin added to usual care reduced cardiovascular death and hospitalisation for heart failure.^6^ Among those with type 2 diabetes and chronic kidney disease, semaglutide reduced by 24% the composite risk of kidney failure, substantial kidney‐function loss, or kidney‐related or cardiovascular death, with additional cardiovascular and survival benefits.^7^ SGLT2 inhibitors and GLP‐1 receptor agonists can therefore prevent events that contribute to disability and long‐term care. However, treatment selection depends on contraindications, tolerability, affordability, and monitoring capacity. Not every patient needs every newer drug, and the expected benefits differ across clinical phenotypes. Outcome‐centred prescribing should match treatment to individual risk, with success assessed through preserved cardiovascular and kidney function, fewer complications, healthier weight, quality of life, and lower treatment burden rather than HbA1c alone.

Long‐term diabetes management demands daily decisions, yet clinic visits provide intermittent support. Continuous glucose monitoring (CGM) reveals glucose fluctuations and responses to food, activity, and treatment, complementing laboratory assessments. Automated insulin delivery (AID) uses CGM data to adjust insulin dosing and may benefit people with type 1 diabetes and insulin‐treated type 2 diabetes. The American Diabetes Association's 2026 Standards of Care emphasise individual needs, preferences, and capacity to use devices.^8^ In a 13‐week randomised trial of 319 adults with insulin‐treated type 2 diabetes, AID reduced HbA1c by an adjusted 0.6 percentage points more than CGM alone and increased time in range by 14 percentage points, with little hypoglycaemia in either group.^9^ Both treatment groups experienced little hypoglycaemia, supporting the clinical promise of AID. Nevertheless, efficacy observed over 13 weeks in trial participants does not establish feasibility or sustained benefit across different healthcare settings. Device costs, digital literacy, alarm burden, connectivity, supplies, and clinical support determine real‐world impact. Integrating remote monitoring with clinical teams allows timely treatment adjustments, education, and complication surveillance. In the SMARTDiabetes cluster trial across 80 Chinese communities, an intervention combining a patient app, family health promoters, and primary‐care decision support increased the proportion achieving at least two of three glycaemia, blood‐pressure, and LDL‐cholesterol targets at 24 months.^10^ Effects varied across rural and urban settings, while blood‐pressure and LDL‐cholesterol outcomes individually did not differ significantly. The SMARTDiabetes findings illustrate how digital interventions can be incorporated into broader care systems, rather than used as standalone applications. In practice, trained teams must interpret incoming data, identify emerging risks, provide sustained follow‐up, and maintain engagement. Technology should strengthen clinical care without increasing the workload borne by patients and families.

Reducing diabetes burden requires coordinated care across the disease continuum. Prevention can delay onset, and early diagnosis can open opportunities for timely and disease‐modifying treatment. Outcome‐centred pharmacotherapy can prevent cardiovascular and kidney events, while technology‐supported care can make long‐term management more responsive. These gains depend on affordability, trained clinical teams, and care models suited to local health‐system capacity. These strategies should be viewed as connected elements of a continuous care pathway, not isolated interventions. Their combined value depends on sustained implementation over years, from prevention and detection to treatment and support in everyday life. Delivering appropriate interventions at each stage can reduce complications, premature mortality, functional decline, and dependence on care. Benefits may also extend to health systems, economies, and society.
